# Supplementary material for: Experimental research on the impact pressure of tidal bore fronts
Source: PLoS One. 2024 May 10;19(5):e0301204. doi: 10.1371/journal.pone.0301204 (PMC11086831; doi:10.1371/journal.pone.0301204)
Supplement: Attachment — Submitted filename: Responses to Reviewers Comments.doc [file pone.0301204.s008.doc]

**Responses to Additional Editor Comments**

**Comment: (1)** The language in the article contains numerous mistakes. These issues significantly hinder the reader's comprehension and should be rectified to enhance the manuscript's overall quality.

**Responses:**We have proofread and corrected writing grammatical issues of the whole manuscript.

**Comment: (2)** List of nomenclature should be included and each abbreviation should be explained.

**Responses:** Each abbreviation had been explained in the revised manuscript.

**Comment:** **(3)** Introduction section is weak and the authors missed the latest relevant research. Most of the literature is outdated. Author should discuss the finding of the earlier research and also discuss the limitations of those studies. The literature study can be enhanced by adding recent relevant references (last five years).

**Responses:** In the revised manuscript, introduction section had been rewrite and the latest relevant research had been added. Findings and limitations of the earlier studies also had been discussed.

**Comment:** **(4)** All the variables should be explained in paper context.
**Responses:** All variables had been explained in the revised paper context.

**Responses to Reviewer #1’s Comments**

We are grateful for the comments of the reviewer, which have greatly helped us to improve the quality of this manuscript. Our responses to the comments are given below.

**Comment:(1)** The flow velocity sensor should also be installed in the test, otherwise the flow velocity cannot be obtained, please add the corresponding description in the test setting.

**Responses:** In the Physical model, we have added the corresponding description in the test setting in lines106-110 and 115-117. “Three capacitive wave-height sensors are equidistantly distributed at 2m in length ways ($\Delta X=2m$). During the bore passage, sensors are used to record the depth of ebb flow ($h$)and the level of tidal bore fronts ($l$), but also they are used to obtain the interval times ($\Delta t_{1}$ and$\Delta t_{2}$) between the three sensors. So$H$could be calculated by$l-h$, and the mean propagation velocity of tidal bore fronts ($U$) could be calculated by $\frac{\Delta X(\Delta t_{1}+\Delta t_{2})}{2\Delta t_{1}\Delta t_{2}}$.

Meanwhile, positioned [parallel](../../../../D:/Program%20Files/Youdao/Dict/6.3.69.8341/resultui/frame/javascript:void(0)%3B) to the second wave-height sensor as shown in Fig 5, an Acoustic Doppler Velocimeter (ADV) was used to measure the velocity of ebb flow($v$), and eight thin film piezoelectric pressure sensors are installed in a 0.015 m diameter Poly Vinyl Chloride (PVC) pipe.”

**Comment:(2)** The h and v used in lines 81 and 82 do not seem to have been defined before using them.
**Responses:** We have made corrections all symbols in the article. Depth of ebb flow represented by$h$while velocities of ebb flow represented by$v$.

**Comment:(3)** Please confirm whether there is any problem with the statement in line 116. This statement looks like it needs polishing.
**Responses:** The statement in line 116 has been rectified more clear. We also have made corrections grammatical mistakes and rectified polishing to enhance the manuscript's statement.

**Comment:(4)** What exactly do the "11 values" in line 71 refer to?
**Responses:** 11 experimental values of height of tidal bore fronts (represented by$H$) were tested between 0.010 m and 0.20 m, such as:when $h\text{ }=\text{ }0.096\text{ }m$ and $v\text{ }=\text{ }0.2\text{ }m/s$, 11 tidal bores with heights from 0.045 m to 0.181 m were measured (Table 1).

**Comment: (5)** The resolution of fig. 1, 3, 5, 6, 7, 8, 11, 12 is too low, please use a higher resolution picture if possible.
**Responses:** All figures have redrawn and get higher resolution pictures.

**Comment: (6)** Fig. 2 Please add the necessary graphic illustration.

**Responses:** Necessary graphic illustrations have added in Fig.2

**Comment: (7)**Redraw Fig. 4, use different colors, or linear lines to distinguish each line, and add legends.
**Responses:** Fig. 4 have redraw using different colors and added legends. It is easily to distinguish each line.

**Comment: (8)** In line 90, please for h and v, and check the full paper.
**Responses:** We checked all symbols and used the correct font.

**Comment:(9)** The full text paragraph format is not uniform, such as 121 lines, please unify the format.
**Responses:** Based on the manuscript body formatting guidelines,we checked and uniformed the full text paragraph format.

**Responses to Reviewer #2’s Comments**

**Comment:(1)**This manuscript contains massive writing issues and should be carefully corrected and improved. Please proofread the whole manuscript and make corrections.

**Responses:** We have proofread and corrected writing issues of the whole manuscript.

**Comment:(2)**The meaning of each letter in mathematical equations should be explained. Please make corrections.

**Responses:** Each letter in mathematical equations of the whole manuscript have been carefully reviewed, explained and corrected.

**Comment:(3)**Mathematical, by replacing Eq.(5) with Eq. (4), the result is not a normal distribution (Eq.6). Please give more details of the derivation of Eq.(6). Please clarify.

**Responses:** The Eq.(6) is not been derived. In the paper , by plotting the non-dimensional $P$ against $Z$in Fig 13, a curve can be obtained. The curve can be seemly described by the probability density function of normal distribution. The Eq.(6) is the form of normal distribution about the curve.

**Comment:(4)**Eq.(11) is a linear equation, and Eq. (10) is a nonlinear equation. A problem arises: why Eq. (10) can be approximated by Eq. (11). Please clarify.

**Responses:** In the paper , we have clarify the meaning of each letter in mathematical equations. Indeed, Eq.(11) is a linear equation and Eq. (10) is a nonlinear equation. But, Eq. (10) is used to calculated the maximum instantaneous pressure value at the elevation z of tidal bore fronts while Eq.(11) is used to calculated the maximum instantaneous pressure of the entire tide fronts at the 0.462times of tidal bore fronts.

**Comment:(5)** The coefficients in Eq.(7) ~Eq.(11) are constant, can they be generalized to the resulting equation and made a derivation?

**Responses:**The coefficients in Eq.(7) ~Eq.(11) are not constant. The height of tidal bore fronts and the elevation of the tidal bore fronts from surface of ebb flow are variable.

**Comment:(6)** The conclusions are ok, and I do not have additional problems.

**Responses:** We are grateful for the comments of the reviewer, which have greatly helped us to improve the quality of this manuscript.

**Comment:(7)** references:  (1) the surname from Ref.1~Ref.14 is incomplete. (2) Ref. 4 and Ref. 6 should add the page number.

**Responses:**Based on the manuscript body formatting guidelines, we have adjusted the cited literature, and corrected all references format uniformed.

**Responses to Reviewer #3****’s Comments**

**Comment:(1)** This review is of “Experimental research on the impact pressure intensity of tidal bore fronts”. Overall I think the results are interesting but I have suggestions for substantial improvement and clarification of motivations and methods. I hope my suggestions are taken constructively and result in an improved manuscript.

**Responses:** We have constructively taken the reviewer’s suggestions. We are grateful for valuable suggestions of the reviewer, which has significantly contributed to the improvement of the quality of this manuscript.

**Comment:(2)** I think the problem the authors are studying—impact intensities from hydraulic bores—is much more relevant and broadly applicable than the authors make it out to be. The topic is very relevant to flash floods, tsunamis, dam break floods, and rogue waves in coastal areas. As the authors themselves say, tidal bores are “limited to only a few locations worldwide”. This is not the way to convince readers of the importance of your research! I recommend emphasizing that the experimental results are applicable to a range of natural phenomena that are important to people and coastal environments, and only then focusing on the narrow case of tidal bores.

**Responses:** We have cited the reviewer’s recommendation.We have rewrite the first paragraph of introduction about tidal bores. We have emphasized tidal bores can be found in various locations around the world and have significant effect on the local ecosystems and human activities. We have add Fig 1.(Photos of the damage situation of a pier (a , b in 1988) and a seawall (c, d in2017) in the Qiantang River) to draw reader’s attention to the strong breaking bore. This is a way to convince readers of the importance of our research.

**Comment:(3)**I recommend adding a simple schematic diagram of the flume instrumentation, including where measurements were done, and that also includes a diagram of a tidal bore showing definitions of variables. For many variables I was not sure where they were measured to or from, and I had to guess.

**Responses:** We have added a schematic diagram of experimental apparatus for generating tidal bores at the downstream end of the flume. We have also added Fig 4 to shown the measurement method and experimental setup for those parameters of tidal bore. Fig 4 includes where measurements were done and definitions of variables.

**Comment:(4)**Finally, I think more details on the pressure measurements are needed. What type of sensor was used? Is it measuring impact forces? Pore pressure? In particular, does it work instantaneously when going from dry to wet? Many pore pressure transduces that I’m familiar with would give numbers, but not really meaningful data, when going from dry to submerged. How quickly do the sensors themselves respond, and what is the sampling rate of recorded data? It is possible that the instrumentation used is appropriate for measuring from dry to wet conditions with impact forces coming from one direction, but the authors should demonstrate that their data can do these things, starting with describing the type of pressure sensors used in the experiments.

**Responses:** The thin film piezoelectric pressure sensor was used for measuring the instantaneous pressure of water flow. It works instantaneously when going from dry to wet. Before data collection, zero calibration is performed in the air and pressure is tested and adjusted right in the water.

In the revised manuscript, we have described the pressure sensor used in the experiments in line 115-121. “Meanwhile, positioned [parallel](../../../../D:/Program%20Files/Youdao/Dict/6.3.69.8341/resultui/frame/javascript:void(0)%3B) to the second wave-height sensor as shown in Fig 5, an Acoustic Doppler Velocimeter (ADV) was used to measure the velocity of ebb flow($v$), while eight thin film piezoelectric pressure sensors are installed in a 0.015 m diameter Poly Vinyl Chloride (PVC) pipe. Sensors are oriented in the direction of the tidal inflow and spaced 0.03 m apart. They are labeled T1 to T8 from bottom to top. Sensors T1 to T3 measure the pressure of ebb flow, while sensors T4 to T8 measure the instantaneous pressure of the tidal bore fronts. Before data collection, zero calibration is performed in the air and pressure is tested and adjusted right in the water. The collected data will undergo further noise reduction processing to obtain pressure of tidal bores. The sampling frequency is 100 Hz.”

**Comment:(5)** Title: I’m not sure the word “intensity” should be in the title, or used in exactly this way. I realize that “intensity” can be used informally or in different ways, but often in physics it is a measure of power, or energy per time. I suggest “Experimental research on impact pressures of tidal bore fronts” or something similar. The authors should consider changing the usage of intensity in other places in the manuscript as well.

**Responses:** We have change all the phrase“the impact pressure intensity”to “the impact pressure” in our paper.We have also revised the title of this paper as “Experimental research on the impact pressure of tidal bore fronts”instead of “Experimental research on the impact pressure intensity of tidal bore front”.

**Comment:(6)** Line 8: suggest changing the English to “Tidal bore impacts can be sufficiently strong and destructive…”
 **Responses:** We have proofread and corrected writing issues of the whole manuscript. We have cited the suggestion and change the sentence to “Tidal bore impact is so sufficiently strong and destructive that estuarine infrastructures are under great threat.”

**Comment:(7)** Lines 33-34, and other places in this and the next paragraph: Don’t just say that a given author studied a topic, say what they found. Descriptions of previous work should give insight or knowledge into the process being studied, not just say that people studied it.
 **Responses:** We have rewrite the introduction about the results of previous research in lines 32~61 and lines67~84. We have not only present the topic of study, but described the method and the result.

**Comment:(8)**33: what is a “model test”? Laboratory experiments? Numerical modeling?
  **Responses:** That is laboratory experiments. We have rewrite the introduction about the results of previous research and recited the new literature, Yeh ’ ariticle have not been cited in the revised manuscript.

**Comment:(9)** 45: cut “preliminarily”; if its published it should no longer be a preliminary result.
 **Responses:** This ariticle had been published, so the word “preliminarily”not correctly used in the initial manuscript. We have deleted it in the revised manuscript.

**Comment:(10)** 66 and below: I think a figure showing from where to where water depth and tidal height are measured is needed, and which direction is considered positive for ebb velocity. How is water depth different from the height of the tidal bore front H? Also, where is z measured (eqn5)?
 **Responses:** We have added a figure (Fig 4.The measurement method for those parameters of tidal bore and ebb flow.) and stated that “the propagation velocity of tidal bore fronts ($U$) is positive and the ebb flow($v$) is negative.” in line132.”

We have also added a figure (Fig 12. Schematic diagram of impact pressure of the tidal bore fronts.) and stated that “$z$is the elevation from water surface of ebb flow at one arbitrary point of the fronts.” in line194.

Water depth means the depth from the water surface of ebb flow, the height of tidal bore(H)means the height from the surface of ebb flow. The depth of ebb flow means the whole depth from the surface of ebb flow.

“Three values of 0.042 m, 0.073 m, and 0.095 m were tested to reflect changes in depth of ebb flow ( the depth from the water surface of ebb flow to the bottom of bed, represented by$h$).”in line102-103.

“Height of tidal bore fronts (the height from the water surface of ebb flow to the water surface of tidal bore fonts, represented by$H$).” in line104-105.

Fig 4.The measurement method for those parameters of tidal bore and ebb flow.

Fig 12. Schematic diagram of impact pressure of the tidal bore fronts.

**Comment:(11)** 70, 71: Variables appear to not show up in the pdf I downloaded; I assume there is supposed to be a variable in “(represented by )”.
 **Responses:** We apologize for our oversight. We have made corrections about the problem of some missing variables.

**Comment:(12)** Figure 2: Its odd to have a figure that mostly shows side-looking ADVs, and then never mention them or saying that you collected data with them but don’t present it here. Is the other device the capacitance-based wave gauge? Consider replacing with a schematic of the pipe containing the pressure probes.
 **Responses:** We have added a figure (Fig 4.The measurement method for those parameters of tidal bore and ebb flow.) and amend a figure(Fig 5. Schematic diagram and photograph of the experimental setup.) .One ADV paralleling with the second wave-height sensors is used for collect the velocity of ebb flow. The others was used for collect the data of tidal bores’ flow field. The data will be used for analyse the turbulence characteristic of tidal bore fronts, which is a topic of our next study.

Fig 5. Schematic diagram and photograph of the experimental setup.

**Comment:(13)**80: Change “front” to “fronts”.
 **Responses:** We have change all the word “front” to “fronts”.

**Comment:(14)**83: I’m not sure what “It is show that the break of the free surface of tidal bore” is trying to say.

**Responses:**This sentence had not state clearly.“the break of the free surface of tidal bore”is meaning of “the form of the tidal bore”. In the revised manuscript,we have change this sentence to “It is shown that the form of the tidal bore is dependent on ....... ”in line 127.

**Comment:(15)**86: Odd to call a relative height of the tidal bore H/h a “strength”. Suggest calling it a relative height or depth. I’m not sure where either H or h are measured without a diagram.
 **Responses:** We have take your advice for changing “strength of tidal bore” to “relative height of the tidal bore”. Meanwhile,the measurement method for those parameters of tidal bore and ebb flow is shown in Fig 4. “During the bore passage, the second sensor are used to record the depth of ebb flow ($h$)and the level of tidal bore fronts ($l$), but also they are used to obtain the interval times $\Delta t_{1}$ and$\Delta t_{2}$ (units of time is second) between the three sensors. So$H$could be calculated by$l-h$, and the mean propagation velocity of tidal bore fronts ($U$) could be calculated by $\frac{\Delta X(\Delta t_{1}+\Delta t_{2})}{2\Delta t_{1}\Delta t_{2}}$. The velocity of ebb flow ($v$) could be recorded by an Acoustic Doppler Velocimeter (ADV) [paralleling](../../../../D:/Program%20Files/Youdao/Dict/6.3.69.8341/resultui/frame/javascript:void(0)%3B) with the second wave-height sensor. ” in lines107-110.

**Comment:(16)**94-95: These two sentences basically say the same thing; change to something like “Figure 5 shows that the three classes of tidal bores vary systematically with Fr and H/h”.

**Responses:** We have change this two sentence to “Through experiments in the flume, forms of the tidal bores have been identified and categorized based on by$Fr$and$H/h$. Fig 8 shows that the three forms of tidal bores vary systematically with $Fr$and$H/h$. ”in lines136-137.

**Comment:(17)**102: Say what aspect of the results are in accord with field data. How are they consistent? What did Xie find?
 **Responses:** In our revised manuscript, we have change this sentence to“Xie et al.^[18,19]^ also observed three forms of tidal bore in the Qiantang River and pointed out the relationship with the Froude number$Fr$. When it exceeds 1.3, the tidal bore surface is no longer smooth. When it is between 1.3 and 1.4, the free surface breaks and forms water roller on the tidal bore fronts. Strong breaking tidal bores occur when it exceeds 1.7. Our results are also consistent with their results of based on field measurements in the Qiantang River. ” in lines142-147.

**Comment:(18)**Equation 2: Give a citation for this equation; if the authors came up with it themselves, make that more clear from the text. I think this is expected for a hydraulic jump.
 **Responses:** Equation 2 is expected for a hydraulic jump. In lines 150-154 of our revised manuscript, “In a horizontal rectangular channel, the relationship between $H/h$ and $Fr$for a hydraulic jump is^[3]^:

$H/h=\eta-1=\text{ }0.5(\sqrt{1+8Fr^{2}}-1)-1$ (2)

where$\eta=(H+h)/h$ is the ratio of hydraulic jump conjugate depth. Here,$H/h$ and $Fr$for hydraulic jumps are cited the same mathematical format as that of tidal bores. Equation (2) is also depicted in Fig 8. It is noteworthy that the curve for hydraulic jumps is an outer envelope of tidal bores. It is seemly like that tidal bore is similar to moving hydraulic jump. ”

**Comment:(19)**114: change tidal to tidal bores;116: change “unit of intensity of pressure” to just “units of pressure”

**Responses:** We had change them in our revised manuscript in line 160 .“Here the units of pressure have been converted from pascals ($Pa$) to meters of water column($mH_{2}O$) by dividing by$\rho g$. ”

**Comment:(20)**Figure 6, also relevant for 7 and 8: I don’t think T1, T2, etc. are defined anywhere. I assume these are different pressure sensors; make that clear and say what heights they were measured at. I can guess that T1 is lowest height and T4 is highest, but just give the reader that information. Also, I think but am not certain that the y axes “l/m” is not saying l divided by m, or per meter, but that “l” in units of meters? Change the notation on all the plots to not use the slash (“/”) to indicate the units, because it usually means divide by. Also, was l ever defined? Is it different from h? Again, a diagram defining variables is needed, as is consistency. Also, I don’t think “t” was ever defined, though I assume the x axis is time in seconds?

**Responses:** “They are labeled T1 to T8 from bottom to top. Sensors T1 to T3 measure the pressure of ebb flow, while sensors T4 to T8 measure the instantaneous pressure of the tidal bore fronts. ” in lines 118-119.

“The level process line collected by the sensor was labeled with the height of tidal bore.The pressure process line collected by the sensor was still labeled with the sensor's label. ” in lines 158-159.

The y axes “*l*/*m*” is not saying *l* divided by *m*, or per meter, but that “*l*” in units of meters. The x axes “*t*/*s*” is not saying *t* divided by *s*, or per second, but that “*t*” in units of second. We redraw figures and corrected the problems of axis titles in all figures.

**Comment:(21)**121-122: This seems to say that the instantaneous pressures are just hydrostatic (increase proportionally with water depth), which seems to be contradicted by the next sentence (123-124) in which pressures increase then decrease as one moves up from the bed. Which is it?

**Responses:** Line 121-122 is describing the the instantaneous pressure of undular bore, but line 123-124 is describing the the instantaneous pressure of weak breaking bore fronts. The instantaneous pressures of Undular bore are similar to hydrostatic (increase proportionally with water depth), but “As shown in Fig 9, the instantaneous pressure of the undular bore fronts is undulating, and the fluctuation amplitude decreases with the increase of water depth. The period of pressure fluctuation is consistent with the water level fluctuation. Fig 10 shows that the instantaneous pressure of weak breaking bore fronts below ebb flow level (T1~T3) is essentially consistent with its water level fluctuation and increases proportionally with water depth. The amplitude of the fluctuation does not significantly differ from that of the tidal level. The instantaneous pressure of weak breaking bore fronts above ebb flow level (T4~T5) exhibit an immediate increase to a maximum value, followed by a rapid decline and accompanied by subsequent fluctuation. Fig 11 shows that instantaneous pressure of strong breaking bore fronts above the ebb flow level (T4~T7) rose to its maximum value before rapid decline. The amplitude of the subsequent fluctuation deviated from the tidal level and exhibited an increasing trend with rising height. Simultaneously, as the height of tidal bore increased, the maximum instantaneous pressure at a given location also rose in tandem with heightened pressure fluctuations. ” in lines167-176.

**Comment:(22)**137: what is the backward fluctuation? I don’t know what this means.

**Responses:** “The backward fluctuation” is our wrong statement. We have corrected it with “the subsequent fluctuation ” in line 172 and 174.

**Comment:(23)**142: what is “pressure pulsation”? I don’t think this was defined.
 **Responses:** “pressure pulsation” is our wrong statement. We have corrected it with “pressure turbulence”in line 179 and 181.

**Comment:(24)**171: Consider giving regression confidence intervals on the parameters in the normal distribution pdf (a, b, c). Change the English from “it can be get that” to “we determine that” or similar.
 **Responses:** We have change the english from “it can be get that” to “we determine that”. in line 207.

**Comment:(25)**174: 0.462 is too precise; suggest just saying 0.5. Its odd to say earlier that the maximum values are between 0.3 and 0.6 (a big range), and then be so specific (0.462) here.
 **Responses:** We have amend the big range in 0.4~0.5and the specific at 0.46.

“Simultaneously, the maximum value of the impact pressure is distributed around 0.4~0.5 times height of tidal bore .” in line 200.

“The maximum impact pressure of strong breaking bore fronts appeared around 0.46 times height of it.” in Abstract and Conclusions.
